# *Hedyotis diffusa* Willd. extract suppresses proliferation and induces apoptosis via IL-6-inducible STAT3 pathway inactivation in human colorectal cancer cells

**DOI:** 10.3892/ol.2015.2956

**Published:** 2015-02-11

**Authors:** JIUMAO LIN, QIONGYU LI, HONGWEI CHEN, HUI LIN, ZIJUN LAI, JUN PENG

**Affiliations:** 1 Academy of Integrative Medicine Biomedical Research Center, Fujian University of Traditional Chinese Medicine, Fuzhou, Fujian 350122, P.R. China; 2 Fujian Key Laboratory of Integrative Medicine on Geriatrics, Fujian University of Traditional Chinese Medicine, Fuzhou, Fujian 350122, P.R. China

**Keywords:** *Hedyotis diffusa* Willd, colorectal cancer, interleukin-6/signal transducer and activator of transcription 3 signaling pathway, apoptosis, proliferation

## Abstract

Recent studies have indicated that the inflammatory microenvironment plays a significant role in colorectal cancer (CRC). The interleukin-6/signal transducer and activator of transcription 3 (IL-6/STAT3) signaling pathway mediates the proliferative and anti-apoptotic activities required for oncogenesis under inflammatory conditions; thus, suppressing tumor growth by targeting the IL-6/STAT3 pathway is a promising therapeutic strategy for CRC. Our previous study reported that the ethanol extract obtained from *Hedyotis diffusa* Willd. (EEHDW) can induce apoptosis, and inhibit the proliferation of colon cancer cells and tumor angiogenesis by modulating various signaling pathways; however, less is known regarding the activity of EEHDW in a cancer-promoting inflammatory environment. Therefore, the present study investigated whether EEHDW inhibits the growth of the CRC HT-29 cell line via the IL-6/STAT3 signaling pathway. Pretreatment of HT-29 cells with IL-6 led to an increase in cell viability, colony formation and phosphorylated STAT3 (p-STAT3) expression. Treatment of these cells with EEHDW prior to IL-6 stimulation resulted in a significant reduction in the IL-6-induced phosphorylation of STAT3. In addition, EEHDW treatment significantly reduced the mRNA expression levels of cyclin D1, cyclin-dependent kinase 4 and B-cell lymphoma-2 (Bcl-2), and upregulated the expression levels of Bcl-2-associated X protein (P<0.05), which are important target genes of the IL-6/STAT3 pathway. These findings strongly indicated that EEHDW suppresses tumor cell growth and induces the apoptosis of human CRC cells via inactivation of the IL-6/STAT3 signaling pathway.

## Introduction

A recent study estimated that >65,000 male and female individuals would likely be diagnosed with colorectal cancer (CRC) in the USA in 2014 (1). With the number of mortalities ranging between 24,000 and 26,000 individuals per year in the USA, CRC is emerging as one of the three leading causes of adult cancer. Various cellular factors have recently emerged as important elements in maintaining the survival and proliferation of CRC tumor cells, including signal transducer and activator of transcription 3 (STAT3) (2). The expression of STAT3 and phosphorylated STAT3 (p-STAT3) has been demonstrated to be significantly higher in CRC tissues compared with healthy intestinal mucosa (3). As STAT3 is activated by numerous growth factors and cytokines, including interleukin-6 (IL-6), the local cytokine environment may have a significant role in the malignancy of CRC (4).

The binding of IL-6 to the IL-6 receptor (IL-6R) initiates an intracellular signaling cascade that activates STAT3 and enhances the localized inflammatory environment, contributing toward c ancer progression (5,6). This association was highlighted by a study that measured the cytokine levels in the sera of CRC patients and identified a direct correlation between IL-6 expression levels and CRC progression (7). Furthermore, activation of the intracellular Janus kinase (JAK)/STAT3 signaling pathway by IL-6 results in the expression of various genes involved in cancer growth and development (8). The phosphorylation of STAT3 in the cytoplasm induces its homodimerization, nuclear translocation and DNA binding (9). p-STAT3 acts as a transcriptional activator of numerous genes, including cyclin D1 and B-cell lymphoma-1 (Bcl-1), with its anti-apoptotic effects significantly contributing to cell proliferation, and tumorigenesis (10,11). Thus, the IL-6/STAT3 pathway is an emerging therapeutic target for CRC.

Standard treatment strategies for CRC include a combination of radiotherapy and chemotherapy, however, the prognosis and survival rates of patients with advanced CRC is poor. The common chemotherapeutic regimens used to treat CRC include 5-fluorouracil (5-FU)/leucovorin, capecitabine, irinotecan, oxaliplatin, bevacizumab and cetuximab (12). In addition to these compounds, various traditional Chinese medicines (TCMs) are currently being evaluated as effective alternatives to the standard chemotherapeutic arsenal. However, the precise mechanism of action of TCMs, as well as the specific pathways that lead to their tumor-suppressive activities, remain unclear.

*Hedyotis diffusa* Willd. [HDW; also known as *Oldenlandia diffusa* (Willd.)] of the Rubiaceae family, is a traditional Chinese herbal medicine that is reported to exhibit a range of pharmacological roles, including anticancer, anti-inflammatory, anti-oxidative, neuroprotective and hepatoprotective activities (13,14). Furthermore, numerous prescriptions of HDW have been demonstrated to provide therapeutic efficacy (15). Our previous studies demonstrated that ethanol extracts obtained from HDW (EEHDW) can induce apoptosis via a mitochondria-dependent pathway in human colon carcinoma HT-29 cells. In addition, treatment with EEHDW appeared to inhibit CRC growth *in vivo* via the inhibition of the STAT3 signaling pathway and suppress tumor angiogenesis via the hedgehog signaling pathway (16–20). Although our previous studies indicated that the activity of EEHDW disrupted the STAT3 pathway, the anticancer efficacy of EEHDW during cytokine-mediated STAT3 activation (such as by IL-6) was largely unclear. Thus, to elucidate the mechanism of the tumoricidal activity of EEHDW, the present study investigated its effects on the IL-6-mediated activation of HT-29 cells *in vitro*. Specifically, cell proliferation and apoptosis, the phosphorylation levels and transcriptional activity of STAT3, and the expression of various target genes of the IL-6/STAT3 signaling pathway were examined to determine the efficacy of EEHDW during cytokine-mediated STAT3 activation.

## Materials and methods

### Materials and reagents

Dulbecco’s modified Eagle’s medium (DMEM), fetal bovine serum (FBS), penicillin-streptomycin, trypsin-EDTA, TRIzol reagent, and caspase-9 and -3 activation kits were purchased from Invitrogen Life Technology, Inc. (Carlsbad, CA, USA). Bcl-2, Bcl-2-associated X protein (Bax), cyclin D1 and cyclin-dependent kinase 4 (CDK4) monoclonal antibodies, as well as horseradish peroxidase (HRP)-conjugated monoclonal secondary antibodies, were obtained from Cell Signaling Technology, Inc. (Danvers, MA, USA). SuperScript II reverse transcriptase was obtained from Promega Corporation (Madison, WI, USA), the DAPI staining kit was obtained from Nanjing KeyGen Biotech Co., Ltd., (Nanjing, China) and the bicinchoninic acid (BCA) protein assay kit was purchased from Tiangen Biotech (Beijing) Co., Ltd. (Beijing, China). All the other chemicals, unless otherwise stated, were obtained from Sigma-Aldrich (St. Louis, MO, USA).

### EEHDW preparation

The HDW plant material was purchased from a commercial supplier (Guo Yi Tang Chinese Herbal Medicine Store, Fujian, China) and the EEHDW was obtained as previously described (20). Stock solutions of EEHDW were prepared by dissolving the EEHDW powder in 40% dimethyl sulfoxide (DMSO) to a final concentration of 400 mg/ml, and stock solutions were stored at −20°C. The working concentrations of EEHDW were made by diluting the stock solution in the culture medium to a final concentration of <0.5% DMSO in the medium.

### Cell culture

Human colon carcinoma HT-29 cells were purchased from the Cell Bank of the Chinese Academy of Sciences (Shanghai, China). Cells were grown as adherent monolayers in DMEM culture media containing 10% (v/v) FBS, 100 U/ml penicillin and 100 μg/ml streptomycin at 37°C in a humidified incubator with an atmosphere of 5% CO_2_.

### EEHDW and IL-6 treatment

The HT-29 cells were cultured with DMEM medium containing 10% FBS and 1% penicillin/streptomycin. When the cells reached ~50% confluency, the complete medium was removed and FBS-free medium was added prior to overnight incubation. The cells were pre-treated with 1, 3 or 5 mg/ml EEHDW in complete DMEM medium for 1 h, followed by stimulation with 10 ng/ml IL-6 for 15 min or 24 h.

### Evaluation of cell viability using an MTT assay

Cell viability was assessed by performing an MTT colorimetric assay. The cells were harvested and resuspended at a final concentration of 1×10^5^ cells/ml, then seeded into 96-well tissue culture plates at a concentration of 100 μl/well. Subsequent to incubating for 24 h at 37°C, the cells were treated with 1, 3 or 5 mg/ml EEHDW and/or 10 ng/ml IL-6 for an additional 24 h. Next, 100 μl MTT (0.5 mg/ml) was added to each well, the plates were incubated at 37°C for 4 h and 100 μl DMSO was added to dissolve the purple formazan crystals. Finally, the absorbance was read at a wavelength of 570 nm using an ELISA reader (Model EXL800; BioTek Instruments, Inc., Winooski, VT, USA).

### Colony formation

The HT-29 cells from exponentially growing cultures were seeded into 12-well culture plates at a density of 1×10^5^ cells/well and were treated with 1, 3 or 5 mg/ml EEHDW and/or IL-6 for 24 h, using the aforementioned protocol. The cells were subsequently harvested and seeded into six-well plates at a final concentration of 1×10^3^ cells/well in 2 ml fresh medium. Following incubation for eight days in a 37°C humidified incubator with an atmosphere of 5% CO_2_, the formed colonies were fixed in MeOH-HAc (v/v dilution, 3:1) for 10 min, stained with crystal violet and counted. The data were normalized to the viability or survival of control cells, set as 100%.

### Cell cycle analysis

A total of 2.5×10^5^ HT-29 cells were seeded into six-well plates in 2 ml medium and treated with 1, 3 or 5 mg/ml EEHDW and/or IL-6 for 24 h. The cells were harvested and adjusted to a concentration of 2×10^5^ cells/ml. Following cell staining with a propidium iodide (PI) cell cycle assay kit, the cell cycle progression of the HT-29 cells was determined using fluorescence-activated cell sorting (FACS). Briefly, the cells were fixed in 70% ethanol at 4°C overnight, then the fixed cells were washed twice with cold PBS, and incubated for 30 min with RNase (8 μg/ml) and PI (10 μg/ml). The fluorescence signal was detected through the FL1 channel of the flow cytometer (FACSCalibur; BD Biosciences, Franklin Lakes, NJ, USA) and the proportion of DNA in various phases of the cell cycle was analyzed using ModFit LT software (version 3.0; Verity Software House, Inc., Topsham, ME, USA).

### Detection of apoptosis by FACS with Annexin V/PI and DAPI staining

A total of 2×10^5^ HT-29 cells were seeded into six-well plates in 2 ml medium and treated with 1, 3 or 5 mg/ml EEHDW and/or IL-6 for 24 h. Subsequently, the apoptosis rate of the HT-29 cells was determined by performing FACS, using a FACSCalibur cell analyzer (BD Biosciences) and an Annexin V-fluorescein isothiocyanate/PI kit, according to the manufacturer’s instructions. In this assay, an Annexin V/PI double-negative population indicates viable cells, and Annexin V-positive/PI-negative or Annexin V/PI double-positive populations represent cells undergoing early or late apoptosis, respectively.

To verify the role of EEHDW in inducing HT-29 cell apoptosis, apoptotic morphology (chromatin condensation and/or nuclear fragmentation) was monitored in DAPI-stained cells. The HT-29 cells were seeded into 12-well culture plates at a density of 1×10^5^ cells/well, and treated with 1, 3 or 5 mg/ml EEHDW and/or IL-6 for 24 h. Subsequently, the cells were washed in PBS, fixed with 4% paraformaldehyde for 10 min and stained with DAPI (4 μg/ml) for 10 min at room temperature. Cover slips containing the cells were washed with PBS and observed under fluorescence microscopy (Leica DMI4000B; Leica Camera AG, Solms, Germany). Cells with clearly defined, condensed nuclei were considered to be apoptotic cells.

### Analysis of caspase-9/-3 activation

The activity of caspase-9 and -3 was determined by performing a colorimetric assay provided in the caspase-9 and-3 activation kit (Invitrogen Life Technologies), in accordance with the manufacturer’s instructions. Briefly, following treatment with 1, 3 or 5 mg/ml EEHDW and/or IL-6 for 24 h, the HT-29 cells were lysed with the provided lysis buffer for 30 min on ice. The lysed cells were centrifuged at 16,000 × g for 10 min and the protein concentration of the clarified supernatant was determined using the BCA assay, according to the manufacturer’s instructions. Subsequently, 100 μg protein was incubated with 50 μl of the specific colorimetric tetrapeptides [Leu-Glu-His-Asp-p-nitroaniline (pNA; specific substrate of caspase-9) or Asp-Glu-Val-Asp-pNA (specific substrate of caspase-3)] at 37°C in the dark for 2 h. Samples were read at a wavelength of 405 nm in an ELISA reader (Model EXL800; BioTek Instruments, Inc.,). The data were normalized to the activity of the caspases in control cells (treated with PBS vehicle) and represented as a fold value of the control.

### Reverse transcription-polymerase chain reaction (RT-PCR) analysis

A total of 2×10^5^ HT-29 cells were seeded into six-well plates in 2 ml medium and treated with 1, 3 or 5 mg/ml EEHDW and/or IL-6 for 24 h. Total RNA was isolated using TRIzol reagent (Invitrogen Life Technologies) and 1 μg oligo(dT)-primed RNA was reverse-transcribed using SuperScript reverse transcriptase (Promega Corporation), according to the manufacturer’s instructions. PCR was performed on the complementary DNA to determine the quantity of cyclin D1, CDK4, Bcl-1 and Bax mRNA, with GAPDH used as an internal control. Samples were analyzed by gel electrophoresis (1.5% agarose) and the DNA bands were analyzed using a Gel Documentation system (Model Gel Doc 2000; Bio-Rad Laboratories, Hercules, CA, USA).

### Western blot analysis

A total of 2×10^5^/ml HT-29 cells were seeded into flasks and pre-treated with 1, 3 or 5 mg/ml EEHDW for 1 h. Subsequently IL-6 stimulation was performed for 15 min for pSTAT3 and STAT3 detection, or 24 h for the analysis of the protein expression of cyclin D1, CDK4, Bcl-2, Bax and Bcl-2. The treated cells were lysed with mammalian cell lysis buffer containing various protein inhibitors and the total protein concentrations were determined by performing a BCA assay. An equal quantity of protein from each cell lysate was subjected to SDS-PAGE and transferred onto polyvinylidene difluoride membranes. The membranes were blocked for 2 h with 5% skimmed dry milk and incubated with the appropriate primary antibody directed against STAT3, p-STAT3, cyclin D1, CDK4, Bcl-2, Bax or β-actin (dilution, 1:1,000) overnight at a temperature of 4°C. Appropriate HRP-conjugated secondary antibodies with chemiluminescence detection were used to image the antibody-detected proteins.

### Statistical analysis

All data summarized in the bar graphs are the mean of three independent experiments, and data were analyzed using the SPSS software package for Windows (version 17.0; SPSS, Inc., Chicago, IL, USA). Furthermore, statistical analysis of the data was performed using Student’s t-test and an analysis of variance. P<0.05 was considered to indicate a statistically significant difference.

## Results

### EEHDW inhibits the growth of HT-29 cells

**Figure 1:**
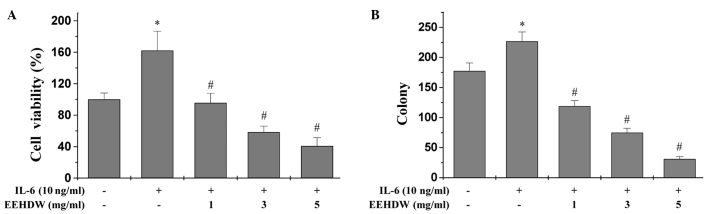
Anti-proliferative activity of EEHDW in HT-29 cells.(A) Effect of three doses of EEHDW on HT-29 cell viability, as assessed by MTT assay. Cells were exposed to EEHDW at the indicated doses for 1 h prior to IL-6 stimulation for 24 h. (B) HT-29 cell proliferation was determined by performing a colony formation assay following EEHDW treatment. The data were normalized to the viability or survival of the control cells. The columns represent the mean of three experiments, and the bars represent the standard deviation from the mean. ^*^P<0.01 vs. controls; ^#^P<0.05 vs. cells treated with IL-6 alone. IL-6, interleukin-6; EEHDW, ethanol extract obtained from *Hedyotis diffusa* Willd.

Our previous study observed that EEHDW reduced the viability and proliferation of HT-29 cells in the absence of IL-6 stimulation (17). To determine whether the potency of EEHDW was maintained under inflammatory conditions, the effect of EEHDW on HT-29 cell viability was measured in the presence of IL-6 by performing an MTT assay (Fig. 1A). IL-6 stimulation appeared to significantly enhance the viability of HT-29 cells by 162.17% compared with the control cells (P<0.05). By contrast, treatment with 1, 3 and 5 mg/ml EEHDW for 24 h reduced the cell viability of the IL-6-stimulated cells in a dose-dependent manner from 95.60 to 40.76% (P<0.05). To determine whether EEHDW was effective at preventing multiple rounds of cell division, the EEHDW-treated HT-29 cells were examined by performing a colony formation assay (Fig. 1B). Treatment with increasing doses (1, 3 and 5 mg/ml) of EEHDW for 24 h significantly reduced the survival rate of the IL-6-stimulated cells by 32.8, 57.6 and 82.5%, respectively (P<0.05).

### EEHDW blocks G_1_/S progression of HT-29 cells

**Figure 2:**
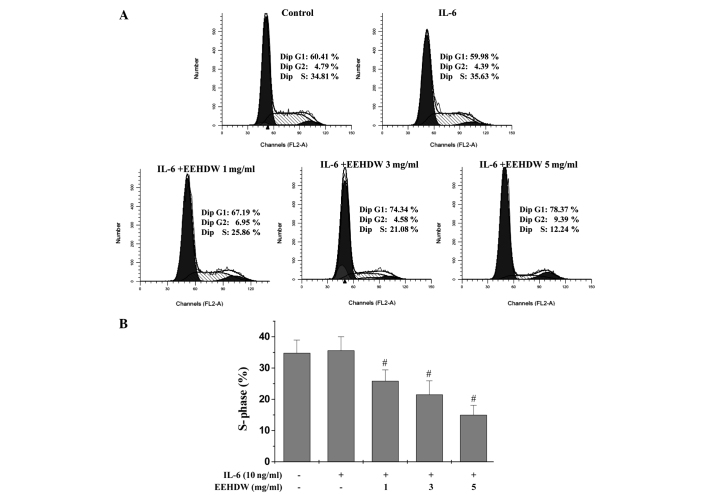
EEHDW inhibits the proliferation of HT-29 cells by blocking G_1_/S cell cycle progression. The cells were pretreated with the indicated doses of EEHDW for 1 h prior to IL-6 stimulation for 24 h. (A) Analysis of cell cycle progression in propidium iodide-stained HT-29 cells by fluorescence-activated cell sorting. The percentage of diploid cells (DNA content) at the G_0_/G_1_, S and G_2_/M phases was quantitated using ModfitLT version 3.0 software. Images are representative of three independent experiments. (B) Comparison of the quantified percentage of HT-29 cells in the S phase based on the various treatment strategies. The columns represent the mean of three experiments, and the bars represent the standard deviation from the mean. ^#^P<0.05 vs. cells treated with IL-6 alone. IL-6, interleukin-6; EEHDW, ethanol extract obtained from *Hedyotis diffusa* Willd.

The G_1_/S transition is one of two major checkpoints that regulate the cell cycle and cell proliferation. Our previous study observed that EEHDW blocked G_1_/S cell cycle progression in the absence of IL-6 stimulation (17). Thus, the present study aimed to investigate whether the effect of EEHDW on IL-6 stimulated HT-29 cells would be similar (Fig. 2A and B). Following staining with PI and FACS analysis, the percentage of S-phase cells was not significantly different between the untreated control and IL-6-stimulated HT-29 cells (34.81 vs. 35.63%, respectively; P>0.05). However, for the HT-29 cells treated with increasing doses of EEHDW (1, 3 or 5 mg/ml), a significant decrease in the percentage of S-phase cells was observed (25.86, 21.08 and 12.24%, respectively; P<0.05). These results indicate that EEHDW inhibits IL-6-stimulated HT-29 proliferation by blocking progression from the G_1_ phase to the S phase of the cell cycle.

### EEHDW induces HT-29 cell apoptosis

**Figure 3:**
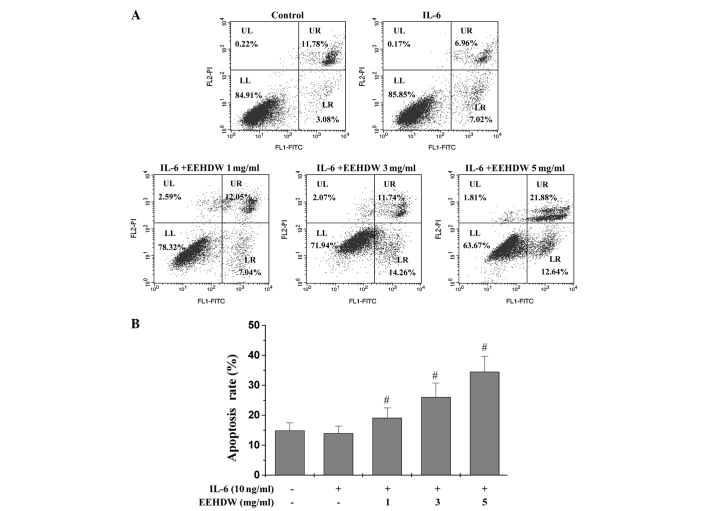
Treatment with EEHDW induces apoptosis in HT-29 cells. Cells were pretreated with the indicated doses of EEHDW for 1 h prior to IL-6 stimulation for 24 h. (A) Fluorescence-activated cell sorting analysis of Annexin V/propidium iodide-stained HT-29 cells. Images are representative of three independent experiments. (B) Comparison of the quantified percentage of early and late apoptotic HT-29 cells based on treatment group. The columns represent the mean of three experiments, and the bars represent the standard deviation from the mean. ^#^P<0.05 vs. cells treated with IL-6 alone. IL-6, interleukin-6; EEHDW, ethanol extract obtained from *Hedyotis diffusa* Willd.; UL, primary necrotic cells; UR, late apoptotic or secondary necrotic cells; LL, viable cells; LR, early apoptotic cells; FITC, fluorescein isothiocyanate.

**Figure 4:**
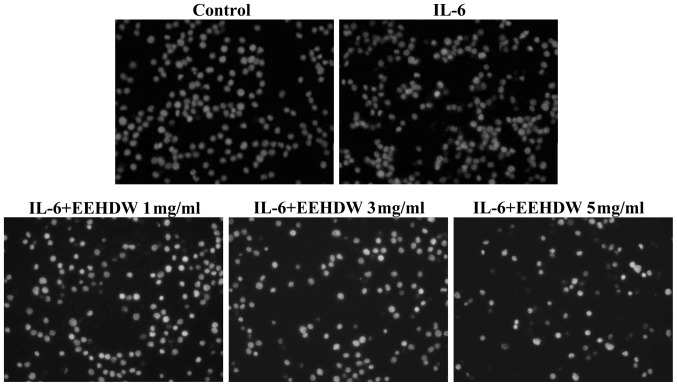
Cellular morphology of EEHDW-treated HT-29 cells. The HT-29 cells were pretreated with the indicated doses of EEHDW for 1 h prior to IL-6 stimulation for 24 h. Changes in cellular morphology were visualized by DAPI staining, and images of the control and treated cells were captured using a phase-contrast fluorescence microscope (magnification, ×200). Images are representative of three independent experiments. IL-6, interleukin-6; EEHDW, ethanol extract obtained from *Hedyotis diffusa* Willd.

Our previous study observed that EEHDW induced apoptosis in HT-29 cells in the absence of IL-6 stimulation (20). To determine whether EEHDEW induces cell apoptosis during cytokine-mediated activation and proliferation, the induction of apoptosis in the IL-6-stimulated HT-29 cells was determined by performing Annexin V/PI staining and FACS analysis (Fig. 3A and B). In comparison to the unstimulated cells, stimulation with 10 ng/ml IL-6 did not significantly alter the proportion of the apoptotic cells (P>0.05). By contrast, EEHDW treatment significantly increased the percentage of cells undergoing early and late apoptosis in a dose-dependent manner (P<0.05 vs. cells stimulated with IL-6 alone). In addition, the cellular morphology and extent of DNA condensation of the apoptotic HT-29 cells were examined by DAPI staining (Fig. 4). Nuclei staining of the HT-29 cells treated with EEHDW was more intense than the untreated cells, indicating that EEHDW promotes HT-29 cell apoptosis in the presence of IL-6 stimulation.

### EEHDW induces the activation of caspases-9 and -3 in HT-29 cells

**Figure 5:**
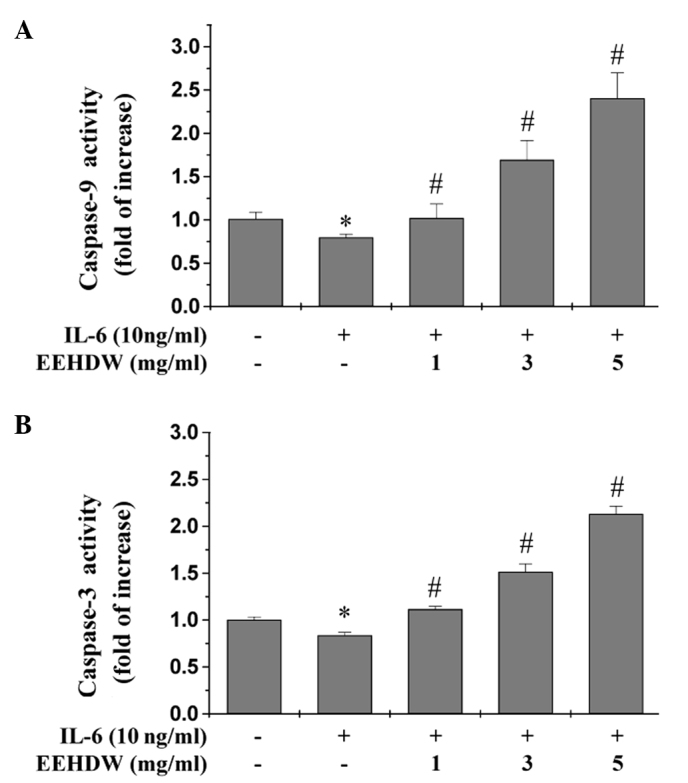
Treatment with EEHDW induces activation of caspases-3 and -9. HT-29 cells were pretreated with the indicated doses of EEHDW for 1 h prior to IL-6 stimulation for 24 h. (A) Caspase-9 and (B) caspase-3 activities were measured using a colorimetric assay, and data were normalized to the mean of the unstimulated control cells (1.0 ng/ml). The columns represent the mean of three experiments, and the bars represent the standard deviation from the mean. ^*^P<0.01 vs. controls. ^#^P<0.05 vs. cells treated with IL-6 alone. IL-6, interleukin-6; EEHDW, ethanol extract obtained from *Hedyotis diffusa* Willd.

Our previous study identified the activation of caspases-9 and -3 in EEHDW-treated HT-29 cells in the absence of IL-6 stimulation (20). Caspases are cytoplasmic, aspartate-specific cysteine proteases whose activation is required for apoptosis, and increased expression of anti-apoptotic factors by the IL-6/STAT3 signaling pathway may reduce caspase-mediated apoptosis in cancer cells (21). As expected, stimulation of the HT-29 cells with IL-6 alone significantly inhibited the activation of caspases-9 and -3 (Fig. 5A and B). By contrast, EEHDW treatment significantly and dose-dependently induced activation of caspases-9 and -3 in the HT-29 cells (P<0.05 vs. the cells stimulated with IL-6 alone; Fig. 5A and B).

### EEHDW inhibits IL-6-mediated STAT3 activation in HT-29 cells

**Figure 6:**
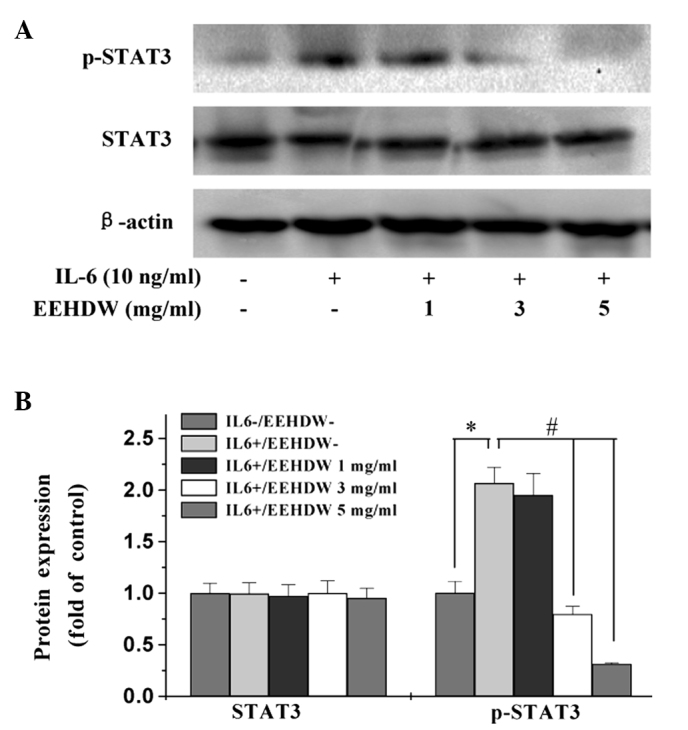
EEHDW treatment inhibits IL-6-mediated STAT3 phosphorylation in HT-29 cells. The HT-29 cells were pretreated with the indicated doses of EEHDW for 1 h prior to IL-6 stimulation for 15 min. (A) Western blot of STAT3 and p-STAT3 in HT-29 cells treated with EEHDW. β-actin served as the loading control (n=3). (B) Densitometric analysis. The data were normalized to the mean protein expression of untreated control. The columns represent the mean of three experiments, and the bars represent the standard deviation from the mean. ^*^P<0.01 vs. controls; ^#^P<0.05 vs. cells treated with IL-6 alone. pSTAT3, phosphorylated signal transducer and activator of transcription; IL-6, interleukin-6; EEHDW, ethanol extract obtained from *Hedyotis diffusa* Willd.

Numerous human cancer cell lines, including HT-29, do not constitutively express p-STAT3 *in vitro*, however, previous studies have demonstrated that IL-6 can stimulate STAT3 activation in HT-29 cells (22). Thus, the present study stimulated STAT3 activation by administering IL-6 to the HT-29 cells, and western blot analysis of the cell lysates was performed to determine the phosphorylation levels of STAT3 at Tyr^705^. Stimulation of the HT-29 cells with IL-6 (10 ng/ml) significantly increased the protein expression levels of p-STAT3, however, phosphorylation was significantly inhibited by EEHDW in a dose-dependent manner (P<0.05) (Fig. 6). By contrast, the protein expression level of non-phosphorylated STAT3 remained unchanged following treatment with IL-6 and/or EEHDW.

### EEHDW significantly downregulates the mRNA and protein expression levels of cyclin D1, CDK4, Bcl-1 and Bax in HT-29 cells

**Figure 7:**
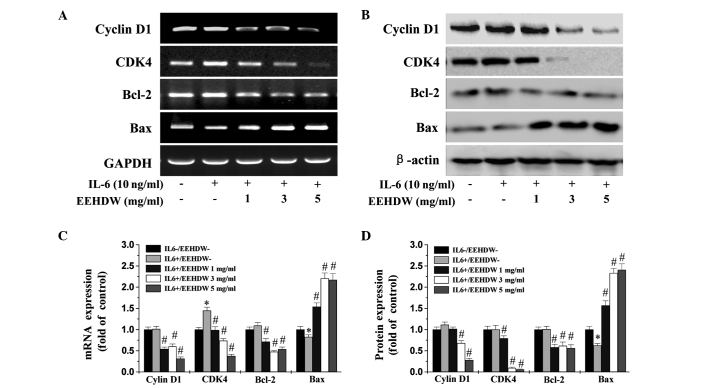
Effect of EEHDW treatment on the mRNA and protein expression levels of Bcl-2, Bax, cyclin D1 and CDK4 in HT-29 cells. The HT-29 cells were pretreated with the indicated doses of EEHDW for 1 h prior to IL-6 stimulation for 24 h. Cell lysates were prepared and assayed for expression of cyclin D1, Bcl-2, Bax and CDK4 by (A) reverse transcription-polymerase chain reaction or (B) western blot analysis. GAPDH and β-actin served as the internal controls for each assay, respectively (n=3). (C-D) Densitometric analysis. The data were normalized to the mean mRNA or protein expression level of untreated control, respectively. The columns represent the mean of three experiments, and the bars represent the standard deviation from the mean. ^*^P<0.01 vs. controls; ^#^P<0.05 vs. cells treated with IL-6 alone. CDK4, cyclin-dependent kinase 4; Bcl-2, B-cell lymphoma-2; Bax, Bcl-2-associated X protein; IL-6, interleukin-6; EEHDW, ethanol extract obtained from *Hedyotis diffusa* Willd.

To investigate the underlying mechanism of action in EEHDW-treated HT-29 cells, RT-PCR and western blot analyses were performed to examine the effect of EEHDW administration on the expression levels of various important target genes of the IL-6/STAT3 signaling pathway. These genes included pro-proliferative cyclin D1 and CDK4, anti-apoptotic Bcl-1, and pro-apoptotic Bax. Excluding CDK4 mRNA expression, the protein and mRNA expression levels of cyclin D1, CDK4, and Bcl-1 were not significantly altered following IL-6 stimulation (P>0.05; Fig. 7). By contrast, EEHDW treatment significantly reduced the IL-6-mediated expression of all three genes at the transcriptional and translational levels (P<0.05). Furthermore, although the mRNA and protein expression levels of Bax were significantly decreased in the presence of IL-6 stimulation (P<0.05), a significant increase in the expression levels of pro-apoptotic Bax were observed in the IL-6-stimulated HT-29 cells treated with various concentrations of EEHDW (P>0.05; Fig. 7).

## Discussion

Specific inhibitors that target a single signaling pathway may be less effective for the treatment of complex tumor systems compared with multi-targeted agents, and the long-term use of multiple single-target-based agents may lead to drug resistance and negative side-effects (23). Although the use of Chinese herbal medicines as an adjunctive therapy for CRC has been widespread in Asia, the efficacy of these treatments has not been well defined. Specific herbal extracts or mixtures within traditional Chinese medicines have demonstrated anticancer properties with fewer side-effects compared with current anticancer treatment strategies, such as chemotherapeutic compounds and antibodies; therefore, recent studies have reexamined the therapeutic potential of traditional herbal medicines (24–26).

Among the cytokines linked to inflammation-associated cancer, IL-6 appears to drive oncogenesis via downstream activation of the JAK/STAT3 signaling pathway. Additionally, dysregulation of the IL-6-mediated JAK/STAT3 signaling pathway is closely associated with the development of a diverse range of solid tumors in humans, including CRC (27,28). Thus, modulation of the IL-6/JAK/STAT3 signaling pathway is currently being analyzed with the aim of developing novel therapies for CRC (29–31). IL-6 is key in promoting cellular proliferation and the inhibition of apoptosis (32), and acts by binding to its receptor (soluble IL-6R) and co-receptor [glycoprotein 130 (gp130)], resulting in activation of the associated Janus kinases (JAKs). Subsequently, the activated JAKs phosphorylate gp130, leading to the recruitment and activation of STAT3 (27), an important transcription factor that is essential in cell survival and proliferation (33,34). Furthermore, overexpression of various genes, including cyclin D1 and Bcl-1, mediated by the abnormal activation of IL-6/STAT3, leads to excessive cell proliferation and apoptotic resistance, which may result in tumorigenesis (8–11).

HDW is a traditional Chinese herbal medicine that exhibits anticancer activities (17,19–21). In the present study, MTT and colony formation assays were used to demonstrate that EEHDW reduces significantly cell viability following IL-6 stimulation (Fig. 1). Although IL-6 stimulation increased the growth of the HT-29 cells, EEHDW treatment significantly increased the number of apoptotic cells in a dose-dependent manner (Fig. 3). Furthermore, the percentage of IL-6-stimulated HT-29 cells in the S-phase significantly decreased compared with the controls cells following treatment with increasing concentrations of EEHDW (Fig. 2). In addition, IL-6 stimulation significantly increased the protein level of pSTAT3; however, phosphorylation of STAT3 was significantly inhibited by the administration of EEHDW in a dose-dependent manner (Fig. 6). Although IL-6 stimulation markedly increased the expression levels of various important target genes of the IL-6/STAT3 pathway, EEHDW treatment significantly reduced IL-6-induced mRNA and protein expression levels of cyclin D1, CDK4, and Bcl-1 (Fig. 7). These data indicate that EEHDW may be a useful therapeutic agent for the treatment of CRC.

In conclusion, HDW is composed of a number of natural products, each of which targets different sites, resulting in the regulation of multiple signaling pathways. The current study provided evidence that the anticancer activity of EEHDW on HT-29 cells acts via the IL-6/STAT3 signaling pathway. However, it remains unknown whether HDW is able to affect other cancer-related signaling pathways, such as mitogen-activated protein kinase, phosphoinositol 3 kinase/Akt and Notch. Therefore, clarification of the molecular mechanisms associated with HDW treatment of cancer is required to develop improved multi-target agents for cancer therapy.
